# LncRNA MT1DP Aggravates Cadmium‐Induced Oxidative Stress by Repressing the Function of Nrf2 and is Dependent on Interaction with miR‐365

**DOI:** 10.1002/advs.201800087

**Published:** 2018-04-24

**Authors:** Ming Gao, Changying Li, Ming Xu, Yun Liu, Min Cong, Sijin Liu

**Affiliations:** ^1^ State Key Laboratory of Environmental Chemistry and Ecotoxicology Research Center for Eco‐Environmental Sciences Chinese Academy of Sciences Beijing 100085 China; ^2^ College of Resources and Environment University of Chinese Academy of Sciences Beijing 100049 China; ^3^ Liver Research Center Beijing Friendship Hospital Capital Medical University Beijing 100050 China; ^4^ Key Labora tory of Ion Beam Bioengineering Hefei Institutes of Physical Science Chinese Academy of Sciences and Anhui Province Hefei Anhui 230031 China

**Keywords:** cadmium, long non‐coding RNA (lncRNA), miR‐365, MT1DP, Nrf2, oxidative stress

## Abstract

Although cadmium (Cd)‐induced hepatoxicity is well established, pronounced knowledge gaps remain existed regarding the inherent cellular signaling that dictates Cd toxicity. Specifically, the molecular basis for determining the equilibrium between prosurvival and proapoptotic signaling remains poorly understood. Thus, it is recently revealed that long non‐coding RNA (lncRNA) MT1DP, a pseudogene in the metallothionein (MT) family, promoted Cd‐induced cell death through activating the RhoC‐CCN1/2‐AKT pathway and modulating MT1H induction. Here, first the dependency of MT1DP induction on MTF1, an important transcriptional factor in driving the mRNA expression of MT1 members is defined. Additionally, a bridge molecule between MT1DP and nuclear factor erythroid 2‐related factor 2 (Nrf2) is established: miR‐365. Mechanistically, MT1DP induction under Cd stress decreases the nuclear factor erythroid 2‐related factor 2 (Nrf2) level to evoke oxidative stress through the elevation of miR‐365, which acted to repress the Nrf2 level via direct binding to its 3'UTR. In contrast to the competing endogenous RNA (ceRNA) mechanism, a new mechanism is proposed: MT1DP elevated the miR‐365 level though stabilizing its RNA via direct binding. Collectively, the combined data demonstrate a crucial role of MT1DP in reducing the Nrf2‐mediated protection of cells, and this is dependent on the interplay with miR‐365. Hence, the study further expands the knowledge of inducible endogenous lncRNA in modulating oxidative stress.

## 1 Introduction

Cadmium (Cd) is recognized to be one of the most common toxic heavy metals in the environment.^1^, ^2^ It is widely dispersed in the atmosphere, water, and soil and can readily enter the food chain, thereby giving rise to a diversity of detrimental health problems.^1^, ^2^ The toxic effects of Cd depend closely on the dose, exposure route, and duration time.^3^ Previous studies have documented that acute Cd exposure causes prominent hepatotoxicity, whereas chronic exposure often leads to renal dysfunction, osteoporosis, anemia, and even cancer.^4^, ^5^ Significant insight has been gained into the molecular basis that underlies Cd‐mediated cytotoxicity, such as oxidative stress‐mediated cellular damage by free radicals (e.g., reactive oxygen species, ROS).^6^, ^7^ Oxidative stress can easily damage cells through lipid peroxidation and can disrupt cellular antioxidant defense systems.^8^, ^9^ Nonetheless, knowledge gaps remain in the understanding of the endogenous cellular machinery that is involved in contributing to Cd toxicity in hepatocytes. Hence, it would be of great interest to shed light on the intracellular machinery that acts against prosurvival signaling under Cd exposure.

Upon initial Cd exposure, cellular antioxidant systems, including enzymatic antioxidants and other prosurvival molecules, are activated to combat the Cd‐induced damage. However, regardless of the activation of these antioxidant systems, a prolonged oxidative stress will eventually disrupt the equilibrium between the proapoptotic and prosurvival signaling, resulting in inevitable cell death.^4^, ^7^, ^10^ Metallothioneins (MTs), which contain abundant thiol groups, are believed to be a crucial protection mechanism against Cd exposure through direct neutralization of the Cd molecule.^4^, ^11^ Nuclear factor erythroid 2‐related factor 2 (Nrf2) is an important antioxidant transcriptional factor and is also activated by Cd to protect the cells against oxidative stress through trans‐activating antioxidative molecules, including hemeoxygenase‐1 (HO‐1) and superoxide dismutase (SOD), among others, via binding to the antioxidant response element.^12^, ^13^ However, in comparison to the prosurvival signaling, much less is known regarding the intracellular proapoptotic signaling. To date, the intracellular proapoptotic molecules that are active in response to Cd and how Cd is implicated in stimulating these proapoptotic molecules remain largely unclear.

Long noncoding RNAs (lncRNAs) are a large cluster of noncoding transcripts containing more than 200 nucleotides. Although lncRNAs are weakly evolutionally conserved across different species and are still poorly characterized, increasing evidence has shown that lncRNAs are implicated in regulating or coordinating various vital biological processes, including cell differentiation, cellular homeostasis, genomic imprinting, and organogenesis and are even involved with diverse pathologies (e.g., cancers).^14^, ^15^, ^16^ In spite of rapid progress,^17^, ^18^, ^19^ the understanding of the molecular mechanisms involved with lncRNAs is still in the early stage. Recent findings have indicated that lncRNAs also serve as part of the cellular antioxidant system that orchestrates signaling pathways to fine‐tune cell death and survival in response to external stresses.^20^, ^21^, ^22^, ^23^

We have recently discovered that a particular lncRNA, MT1DP, functions to shunt the cellular defense to cytotoxicity through crosstalk with MT1H and RhoC under cadmium stress in hepatocytes.^24^ The primary objective of the current study is to further define the role of MT1DP in reinforcing Cd toxicity in hepatocytes. Here, we aimed to determine whether MT1DP responded to Cd exposure and had a function in the regulation of the Cd‐induced signaling network. Taken together, our results revealed a novel role of MT1DP in provoking Cd toxicity by diminishing Nrf2 through stabilizing its negative regulator, miR‐365.

## 2 Results

### 2.1 MT1DP Expression is Subject to Cd‐Induced Oxidative Stress

**Figure 1:**
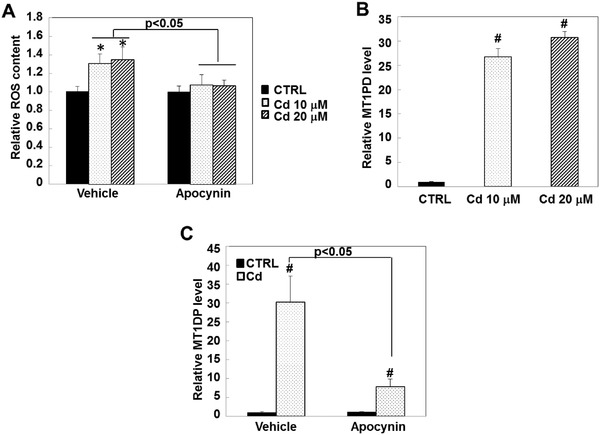
MT1DP is regulated by Cd‐induced ROS production. A) HepG2 cells were treated with or without apocynin for 1 h prior to the indicated dosage of Cd for 6 h; the ROS contents were determined by multiscan spectrometry (*n* = 6) (*p < 0.05, relative to untreated cells). B) The expression level of MT1DP in liver cells in response to 10 and 20 µm Cd were analyzed by qRT‐PCR (*n* = 3) ^#^ *p* < 0.001, relative to the untreated cells). C) HepG2 cells were pretreated with or without apocynin at 100 µm for 1 h prior to treatment with 20 µm Cd for 6 h; the MT1DP level was analyzed by qRT‐PCR (*n* = 3) (^#^ *p* < 0.001, relative to the untreated cells).

We recently showed that the lncRNA MT1DP was a crucial regulator in promoting Cd‐induced cell death.^24^ However, whether MT1DP induction is subject to Cd‐induced oxidative stress has remained unclear. To address this question, we determined MT1DP levels under various endogenous oxidative stress conditions. As shown in **Figure** **1**A,B, intracellular oxidative stress, as reflected by cellular ROS content, was significantly increased by more than 30% under Cd treatment at 10 and 20 µm for 6 h (*p* < 0.05). Likewise, the expression levels of MT1DP were increased by more than 20‐fold under Cd treatment at 10 and 20 µm for 6 h relative to untreated cells (*p* < 0.001), which is suggestive of a close correlation between Cd‐triggered oxidative stress and MT1DP induction. When Cd‐induced ROS production was bleached by pretreatment with the NADPH‐oxidase inhibitor, apocynin (Figure 1A, *p* < 0.05), the induction of MT1DP expression was analogously compromised by 70% compared with the control cells in response to 20 µm Cd (Figure 1A,C, *p* < 0.05). These data indicated that MT1DP induction by Cd treatment is, at least in part, subject to oxidative stress.

### 2.2 MTF1 Dictates MT1DP Expression at the Transcriptional Level upon Cd Exposure

**Figure 2:**
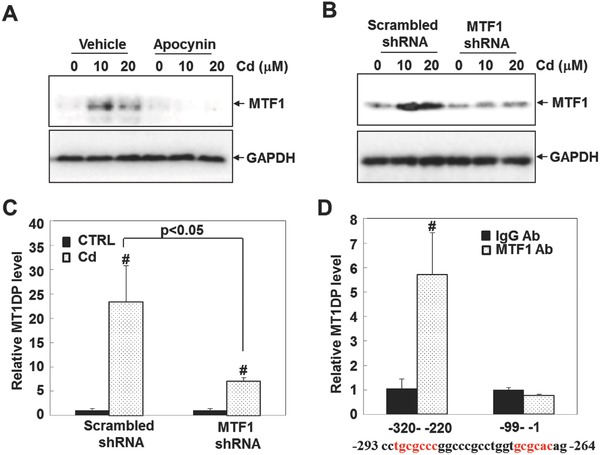
MTF1 regulates MT1DP expression at the transcriptional level. A) HepG2 cells were first pretreated with or without apocynin for 1 h prior to 10 and 20 µm Cd for 6 h, and the MTF1 protein content was determined by Western blot analysis. B) Western blot analysis of MTF1 concentrations in the scrambled control and MTF1‐knockdown cells in response to the indicated doses of Cd for 6 h. C) Expression levels of MT1DP were detected in the scrambled control and MTF1‐knockdown cells upon 20 µm Cd treatment using qRT‐PCR analysis (*n* = 3) (^#^ *p* < 0.001, compared to untreated cells). D) Chromatin immunoprecipitation assay was performed using an antibody against MTF1 to pulldown the indicated sequence of MT1DP, followed by qRT‐PCR analysis (*n* = 3) (^#^ *p* < 0.001, relative to the normal IgG control).

Given that the MT1 subfamily members with metal response elements (MREs) can be recognized and activated by metal‐responsive transcription factor 1 (MTF‐1),^25^, ^26^ we hypothesized that MT1DP might also be a downstream target of MTF1 in response to Cd. As shown in **Figure** **2**A, similar to MT1DP, the MTF1 level was also driven by the oxidative stress under Cd treatment, as Cd treatment increased the MTF1 level and apocynin pretreatment reduced its level (Figure 2A). To clarify whether MT1F was responsible for the expression of MT1DP, the induction of MT1DP was determined in the cells with endogenous MTF1 knockdown. As shown in Figure 2B, the MTF1 concentration was greatly reduced in cells with shRNA‐mediated gene silencing, with or without Cd treatment. Although Cd could still markedly promote MT1DP induction in the MTF1‐knockdown cells (*p* < 0.001), the induction of MT1DP levels in MTF1‐knockdown cells was much weaker (an ≈70% reduction) than that in the scrambled control cells responding to Cd stress (Figure 2C, *p* < 0.05). These findings, therefore, suggest that MT1DP is a downstream target of MTF1.

To test this assumption, subsequent bioinformatic analysis revealed a region with two putative MREs within the promoter region of MT1DP (Figure 2D, lower panel). To corroborate a direct binding of MTF1 to the region containing these MREs, a chromatin immunoprecipitation assay was carried out. As shown in Figure 2D, MTF1 antibody (Ab) greatly enriched the region with the putative MREs (−320 to −220 in the promoter) by nearly 6‐fold in comparison to normal IgG (*p* < 0.001), and a reference region (−99 to −1 in the promoter) without the putative MREs was used as the negative control. These results demonstrate that MT1DP is directly regulated by MTF1 through the MREs at the transcriptional level upon Cd treatment.

### 2.3 MT1DP Induction Aggravates Cd Toxicity

**Figure 3:**
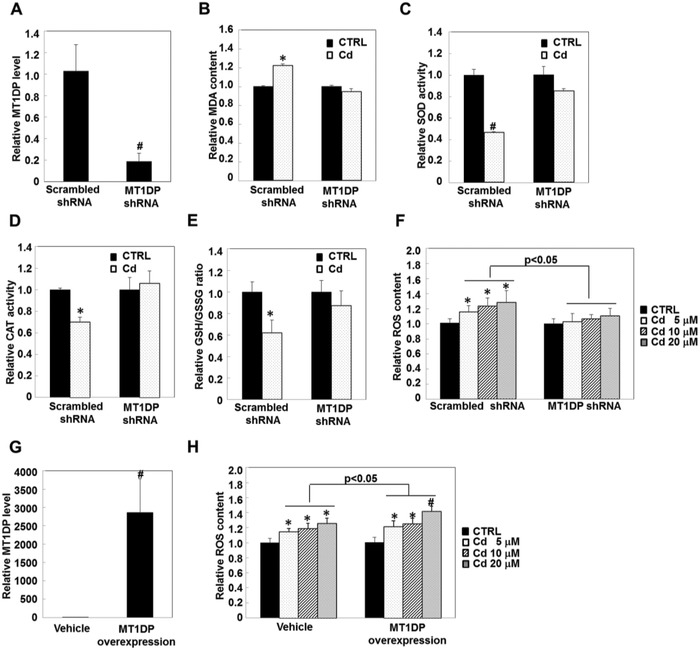
MT1DP plays an important role in regulating Cd‐induced oxidative stress. A) Expression levels of MT1DP in the scrambled control and MT1DP‐knockdown cells were determined by qRT‐PCR assay (*n* = 3) (^#^ *p* < 0.001). B–E) The scrambled control and MT1DP‐knockdown cells were treated with 20 µm Cd for 6 h, and the cellular MDA content, SOD, and CAT activities, and the GSH/GSSG ratio were assayed (*n* = 4) (*p < 0.05 and ^#^ *p* < 0.001, compared to untreated control). F) The production of ROS content was measured in the scrambled control and MT1DP shRNA cells responding to Cd at various concentrations for 6 h by multiscan spectrometry (*n* = 6) (*p < 0.05, compared to the untreated cells). G) Expression levels of MT1DP were determined in vehicle control and MT1DP overexpression cells by qRT‐PCR assay (*n* = 3) (^#^ *p* < 0.001). H) The production of ROS content was assessed in vehicle control and MT1DP overexpression cells in response to the indicated doses of Cd for 6 h by multiscan spectrometry (*n* = 6) (*p < 0.05 and ^#^ *p* < 0.001, compared to the untreated cells).

To understand the physiological function of MT1DP induction upon Cd exposure, we used a shRNA strategy to knock down the endogenous MT1DP by ≈80% in the HepG2 cells (**Figure** **3**A, *p* < 0.001) and then compared the cellular oxidative status between the control cells and the MT1DP‐knockdown cells upon Cd treatment. As shown in Figure 3B, Cd induced an increase of malondialdehyde (MDA), a hallmark of oxidative stress, in the control cells (122% induction relative to the untreated cells, *p* < 0.05), whereas no significant change of MDA content was observed in the MT1DP‐knockdown cells. To confirm the role of MT1DP in regulating the cellular redox status under Cd treatment, the antioxidant indicators SOD, catalase (CAT), and the glutathione/glutathione disulfide (GSH/GSSG) ratio were also determined. As presented in Figure 3C–E, when MT1DP was knocked down, the decrease in the SOD and CAT activities and the GSH/GSSG ratio in response to Cd were all markedly reversed (*p* < 0.05), signifying that MT1DP is a novel regulator of Cd‐induced oxidative stress. Moreover, dichlorofluorescein diacetate (DCF‐DA) dye was also used to investigate the effect of MT1DP on Cd‐induced ROS production. As shown in Figure 3F, Cd‐induced dose‐dependent ROS production was also markedly attenuated in the MT1DP‐knockdown cells (*p* < 0.05), indicating the important role of MT1DP in evoking oxidative stress in response to Cd. To substantiate this finding, the MT1DP content was augmented by the transfection of an MT1DP overexpression construct (Figure 3G). As a result, the Cd‐associated dose‐dependent ROS generation was further enhanced by 6–13% relative to the Cd‐treated scrambled control cells (Figure 3H, *p* < 0.05). These data revealed that MT1DP aggravates Cd‐induced oxidative stress.

### 2.4 MT1DP Represses Nrf2 Levels to Enhance ROS Accumulation upon Cd Treatment

**Figure 4:**
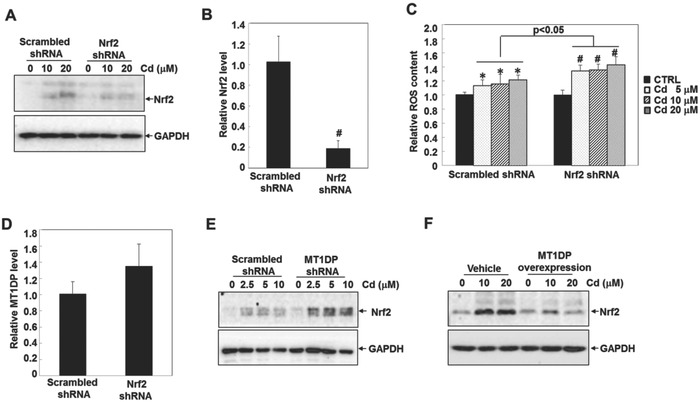
Nrf2 attenuates Cd‐induced oxidative stress. A) The scrambled control and Nrf2‐knockdown cells were treated with 10 and 20 µm Cd for 6 h; the protein contents of Nrf2 were then determined by Western blot analysis. B) Expression levels of Nrf2 were assessed in the scrambled control and Nrf2‐knockdown cells by qRT‐PCR assay (*n* = 3) (^#^ *p* < 0.001). C) ROS contents in the scrambled control and Nrf2‐knockdown cells were measured by multiscan spectrometry in response to the indicated doses of Cd for 6 h (*n* = 6) (*p < 0.05 and ^#^ *p* < 0.001, compared to the untreated cells). D) Expression levels of MT1DP were compared in the scrambled control and Nrf2‐knockdown cells by qRT‐PCR assay (*n* = 3). The protein concentrations of Nrf2 E) in the scrambled control and MT1DP‐knockdown cells, and F) in vehicle control and MT1DP overexpression cells were analyzed by Western blot.

Next, we attempted to delineate the molecular basis underlying MT1DP‐dependent ROS promotion. As a master regulator, Nrf2 functions to combat oxidative stress‐associated cellular injury through diverse mechanisms, including removal of free radicals via the induction of antioxidant components, such as HO‐1 and SOD1.^27^, ^28^ In particular, Nrf2 has been shown to play a critical role against Cd‐induced hepatotoxicity.^13^ Here, we endeavored to elucidate the potential regulation of Nrf2 by MT1DP. Consistent with previous studies,^29^ Cd treatment significantly increased Nrf2 concentrations in the HepG2 cells (**Figure** **4**A). This concentration increase upon the addition of Cd could be strongly compromised in the cells with Nrf2 knockdown (Figure 4A,B). In agreement with previous findings,^30^ Nrf2 knockdown enhanced Cd‐induced dose‐dependent ROS generation by 15–18% compared to the control cells (Figure 4C, *p* < 0.05), which highlights the important role of Nrf2 in combating Cd‐induced oxidative stress.

To identify the link between Nrf2 and MT1DP, we next investigated the alterations of MT1DP and Nrf2 upon their mutual knockdown. As shown in Figure 4D, Nrf2 knockdown had no significant effect on the expression of MT1DP. However, Cd‐induced Nrf2 accumulation could be enhanced in the cells with a reduction in MT1DP (Figure 4E). Meanwhile, the induction of Nrf2 by Cd was significantly reversed in cells with MT1DP overexpression (Figure 4F). These observations, therefore, reveal the regulation Nrf2 by MT1DP, particularly under Cd‐induced oxidative stress.

### 2.5 miR‐365 is the Intermediate Downstream of MT1DP That Decreases Nrf2

**Figure 5:**
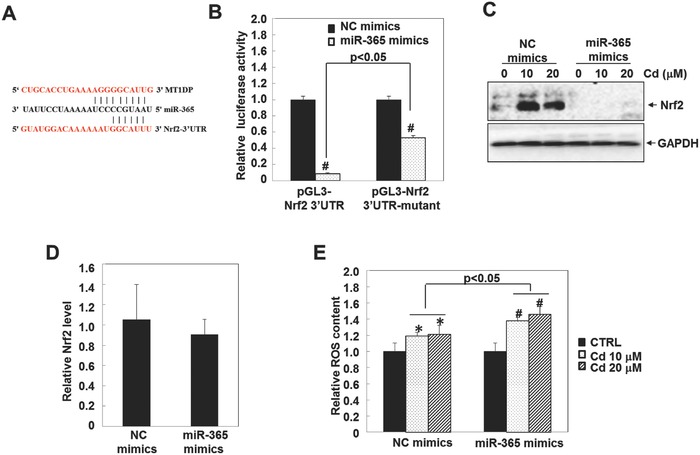
MT1DP regulates Nrf2 expression via miR‐365. A) A schematic illustration of the binding site of miR‐365 within the MT1DP and Nrf2 3'UTR regions. B) Cells with pGL3‐Nrf2 3'UTR and pGL3‐Nrf2 3'UTR‐mutant construct overexpression were transfected with NC‐mimic or miR‐365 mimic molecules for 24 h; the relative luciferase activities were then measured by a dual‐luciferase assay (*n* = 3) (^#^ *p* < 0.001). C) HepG2 cells were transfected with NC‐mimic and miR‐365 mimic molecules for 24 h prior to Cd exposure for 6 h; the Nrf2 protein content was then detected by Western blot. D) The mRNA levels of Nrf2 were detected in the cells transfected with NC‐mimics and miR‐365 mimics by qRT‐PCR assay (*n* = 3). E) Cells transfected with NC‐mimics and miR‐365 mimics were treated with Cd for 6 h; ROS production was then determined by multiscan spectrometry (*n* = 6) (*p < 0.05 and ^#^ *p* < 0.001).

To interpret the mechanism of how MT1DP regulates the Nrf2 level, we searched for partner molecules that could bind both MT1DP and Nrf2. As shown in **Figure** **5**A, MT1DP and Nrf2 3'UTR share a consensus binding site on miR‐365, suggestive of a regulation mechanism among these three RNA molecules. To investigate, we cloned the 3'UTR of Nrf2 mRNA into a luciferase reporter to test the impact of miR‐365 on Nrf2 expression. As shown in Figure 5B, the relative luciferase activity of pGL3‐Nrf2‐3'UTR markedly declined by 90% upon the application of miR‐365 mimics (*p* < 0.05). However, when the binding site of miR‐365 within the 3'UTR of Nrf2 was mutated, the inhibitory effect of miR‐365 on the activity of Nrf2‐3'UTR was also significantly compromised (Figure 5B, *p* < 0.05). Similarly, Cd‐induced Nrf2 protein accumulation was also effectively inhibited by miR‐365 mimics (Figure 5C), further confirming that Nrf2 is directly modulated by miR‐365, even under Cd treatment. Moreover, the mRNA level of Nrf2 was practically unchanged when miR‐365 was overexpressed (Figure 5D), suggesting that miR‐365 regulated the concentration of Nrf2 at the translational level. Importantly, the cellular ROS generation upon Cd treatment was further enhanced by 15% and 20% in the HepG2 cells upon transfection of miR‐365 mimics under Cd treatment at 10 and 20 µm, respectively (Figure 5E, *p* < 0.05). These results show that miR‐365 serves as an intermediate between MT1DP and Nrf2, playing an important role in promoting Cd‐induced oxidative stress by suppressing Nrf2 concentrations at the translational level.

### 2.6 MT1DP Enhances the RNA Stability of miR‐365

**Figure 6:**
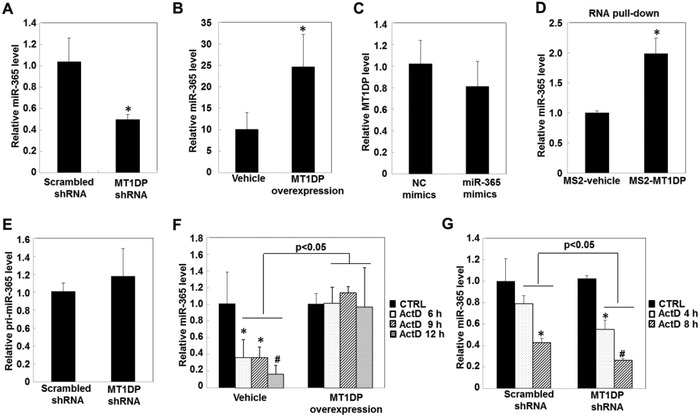
MT1DP interacts with and stabilizes miR‐365. The miR‐365 level in A) the scrambled control and MT1DP‐knockdown cells, and B) in vehicle control and MT1DP overexpression cells, assessed by qRT‐PCR (*n* = 3) (*p < 0.05). C) Expression levels of MT1DP in cells transfected with NC‐mimics and miR‐365 mimics were analyzed by qRT‐PCR (*n* = 3). D) Relative enrichment of miR‐214 was assessed through an RNA pull‐down assay in cells transfected with MS2‐vehicle and MS2‐MT1DP constructs (*n* = 3) and was then detected by qRT‐PCR (*p < 0.05). E) Expression levels of pri‐miR‐365 were detected in the scrambled control and Nrf2‐knockdown cells by qRT‐PCR. F) The scrambled control and MT1DP‐knockdown cells and G) vehicle control and MT1DP overexpression cells were treated with 5 µm ActD for the indicated time; the expression levels of miR‐365 were then measured by qRT‐PCR (*n* = 3) (*p < 0.05 and ^#^ *p* < 0.001, relative to the untreated cells).

Of interest is to clarify how MT1DP modulates miR‐365 to enable the latter's action on Nrf2. To answer this question, we investigated the regulation of MT1DP on miR‐365. As shown in **Figure** **6**A, the miR‐365 level was significantly reduced by 50% upon MT1DP knockdown in MT1DP shRNA cells compared to that of the scrambled control cells (*p* < 0.05). In contrast, the miR‐365 level was increased by 150% in the MT1DP overexpression cells relative to that in the vehicle control cells (*p* < 0.05). Notably, miR‐365 mimics had no significant effect on the level of MT1DP (Figure 6C), ruling out a mutual regulation between miR‐365 and MT1DP.

To understand the mechanism underlying the regulatory activity of MT1DP on the miR‐365 level, an RNA‐pull down assay was performed by using MS2 hairpin‐tagged MT1DP to elucidate whether MT1DP could directly bind to miR‐365. As shown in Figure 6D, miR‐365 was more highly enriched in MS2‐MT1DP pulldown cell lysate compared to that in the MS2‐vehicle control cells (*p* < 0.05), pointing to a direct interplay interaction. In addition, MT1DP deficiency elicited no effect on the expression of pri‐miR‐365 (Figure 6E), indicating that MT1DP did not regulate miR‐365 at the transcriptional level. To further understand the molecular basis for the control of MT1DP on miR‐365, an inhibitor of DNA transcription and replication, dactinomycin/actinomycin D (ActD), was used to block the transcription of miR‐365. As shown in Figure 6F, the endogenous expression level of miR‐365 dropped gradually over the time course from 6 to 12 h upon treatment with ActD (*p* < 0.05); however, the decline of miR‐365 was markedly blocked by the overexpression of MT1DP in the ActD‐treated cells (*p* < 0.05). In contrast, the decline rate of miR‐365 was further accelerated in the ActD‐treated cells upon MT1DP knockdown (Figure 6G, *p* < 0.05). Together, our data demonstrate that MT1DP functions to elevate the miR‐365 concentration by enhancing miR‐365 RNA stability.

## 3 Discussion

MT1DP is a pseudogene that belongs to the MT1 subfamily and is specifically present in human genome.^31^, ^32^ Previous studies have suggested that MT1DP acts as a tumor suppressor to promote liver cell apoptosis through regulating YAP and Runx2.^33^ However, the biological functions of MT1DP are still largely unknown. Our recent results have shown that the MT1DP promotes Cd‐induced cell death through calibrating the activation of the RhoC‐CCN1/2‐AKT signaling pathway and modulates the induction of MT1H.^24^ However, the role of MT1DP in dictating Cd‐induced cytotoxicity has remained unclear. For example, the molecular link to oxidative stress has not been fully elucidated. Therefore, in this study, we have continued our investigation of these mechanisms. We first defined the dependency of MT1DP induction by Cd on MTF1, which is the critical transcriptional factor that drives mRNA expression for protein‐coding MT1 subfamily members. More importantly, a major finding in this study is clarifying the link between MT1DP to Nrf2 through a bridge molecule, miR‐365. Nrf2 is a master regulator that drives the expression of many antioxidant and detoxification enzymes.^12^ Nrf2 protein induction is an adaptive response to Cd‐induced oxidative stress, removing intracellular ROS.^13^, ^34^ Thus, Nrf2 deficiency would considerably potentiate the oxidative stress‐associated injury to cells due to inadequate levels of antioxidant and detoxification enzymes.^30^, ^35^ Our results revealed that MT1DP induction by Cd negatively regulates the Nrf2 concentration to evoke oxidative stress, and, importantly, we have defined the downstream intermediate of MT1DP, miR‐365, which acts to repress Nrf2 levels.

It has been reported that several miRNAs, such as miR‐144, miR‐28, and miR‐153, can negatively regulate Nrf2 levels by directly targeting the 3'UTR of Nrf2 mRNA.^36^, ^37^ Here, miR‐365 was identified as a novel miRNA that decreases Nrf2 levels by binding to its 3'UTR. The majority of previous studies in this area have shown that lncRNAs can govern the level of protein coding transcripts through a competing endogenous RNA (ceRNA) mechanism, which is dependent on its partner miRNAs.^18^ In contrast to this established principle, here we have defined a new mechanism: MT1DP stabilizes miR‐365 by direct binding, which in turn enhances the function of miR‐365 to repress Nrf2 concentrations at the post‐transcriptional level. Additionally, miR‐365 was shown to have no impact on MT1DP, highlighting a novel regulation of RNA stability for miR‐365 by MT1DP.

In summary, the current study has demonstrated an important role of MT1DP in aggravating oxidative stress in hepatocytes upon Cd treatment through its interplay with miR‐365, which functioned as the intermediate to repress Nrf2‐mediated antioxidant signaling. Specifically, MTF1 was found to be induced by Cd in order to activate MT1DP expression in the hepatocytes; MT1DP, in turn, elevated the miR‐365 level through a protection mechanism, dependent on the direct binding between them. Consequently, miR‐365 decreased Nrf2 levels, provoking Cd‐induced oxidative stress. Our results reveal a new mechanism underlying the MT1DP‐conducted signaling that promotes Cd toxicity in the hepatocytes.

## 4 Experimental Section

*Cell Culture*: The human hepatocellular carcinoma cell line HepG2 was obtained from the Cell Resource Center of the Institute of Basic Medical Sciences (CAMS, China). Cells were grown in Dulbecco's modified Eagle medium (DMEM) (Hyclone, Logan, UT, USA) with 10% fetal bovine serum (HyClone) and penicillin/streptomycin (HyClone) at 37 °C with 5% CO_2_.

*Cell Transfection*: miRNA mimics were purchased from Genepharma Biotechnology (Shanghai, China). The pcDNA3.1‐MT1DP, MT1DP shRNA, and MS2‐MT1DP were constructed as previously described.^24^ The human MTF1 and Nrf2 shRNA sequences were cloned into the vector PLKO.1 to construct the MTF1 shRNA and Nrf2 shRNA plasmids. The Nrf2 3'UTR sequence was amplified and cloned into the luciferase reporter vector PGL3‐promoter to obtain pGL3‐Nrf2 3'UTR transfectant. Oligonucleotides and plasmids were transfected into cells using Lipofectamine 2000 transfection reagent (Invitrogen, Carlsbad, CA, USA) according to the manufacturer's instruction.

*Western Blot Analysis*: HepG2 cells were lysed with radio immunoprecipitation assay (RIPA) buffer (Solarbio, Beijing, China) with protease inhibitor cocktail (Roche, Basel, Switzerland) using a standard method. After mass quantification using the Bradford protein concentration assay (Solarbio, Beijing, China), the cell extracts were separated by sodium dodecyl sulfate polyacrylamide gel electrophoresis and then transferred onto the nitrocellulose membranes (Millipore, Billerica, MA, USA), as described previously.^38^, ^39^ MTF1, Nrf 2, and GADPH primary Abs were purchased from Proteintech Group (Wuhan, China).

*Luciferase Reporter Assay*: Cells were harvested 48 h after transfection and luciferase activity was measured in the form of chemiluminescence using the dual‐luciferase reporter assay system (Promega, Medison, MI, USA) following the manufacturer's instructions. The relative luciferase activity was normalized to the according Renilla luciferase activity.

*Real‐Time PCR (qRT‐PCR)*: Total RNA was extracted by Trizol reagent (Invitrogen), and first strand cDNA was synthesized using the Thermo script RT‐polymerase chain reaction (PCR) system (Thermo scientific, Waltham, MA, USA) according to the manufacturer's instructions. qRT‐PCR was performed on an iQ5 PCR instrument (Bio‐Rad, Hercules, CA, USA), as described previously.^38^, ^39^ The primer sequences are listed in **Table** **1**.

**Table 1:** Primers used in the current study

Primers for shRNA construction	Sequences (5′‐3′)
MT1DP shRNA F	ccggaatgcaaagagtacaaatgcactcgagtgcatttgtactctttgcatttttttg
MT1DP shRNA R	aattcaaaaaaatgcaaagagtacaaatgcactcgagtgcatttgtactctttgcatt
Nrf2 shRNA F	ccggccggcatttcactaaacacaactcgagttgtgtttagtgaaatgccggtttttg
Nrf2 shRNA R	aattcaaaaaccggcatttcactaaacacaactcgagttgtgtttagtgaaatgccgg
Primers for plasmid construction	Sequences (5′‐3′)
pGEMT‐MT1DP F	cctgtggcttaggaactccagcctcacctg
pGEMT‐MT1DP R	taaggccaacaatgtttattatc
MSP‐MT1DP F	gccgatatcatagaacatccctggggcagg
MSP‐MT1DP R	tgctctagaaaggccaacaatgtttattatcattcc
pGL3‐Nrf2 3′UTR F	cgagctcatttaggaggatttgaccttttctg
pGL3‐Nrf2 3′UTR R	ccgctcgagatttaacagtcataataatcctt
pGL3‐Nrf2 3′UTR mutant F	tactgtatggacaaaaaatgacgtgttttatataaattgtttagctc
pGL3‐Nrf2 3′UTR mutant R	gagctaaacaatttatataaaacacgtcattttttgtccatacagta
Primers for qRT‐PCR	Sequences (5′‐3′)
MT1DP F	tcaaggccaaaggtggctcctgcac
MT1DP R	gcacggcagctgcacttcaccaatg
GAPDH‐F	gaaggtgaaggtcggagt
GAPDH‐R	gaagatggtgatgggatttc
U6 sn F	ctcgcttcggcagcaca
U6 sn R	aacgcttcacgaatttgcgt
miR‐365 loop	ttaactggatacgaagggtccgaacaccggtcgtatccagttaaataaggat
miR‐365 F	tgcggtaatgcccctaaaaa
miR‐365 R	tacgaagggtccgaacac
Nrf2 F	tccagtcagaaaccagtggat
Nrf2 R	gaatgtctgcgccaaaagctg
pri‐miR 365 F	accgcagggaaaatgaggg
pri‐miR 365 R	tgcaagagcaataaggatt

*Antioxidant Components Determination*: The cells were treated with or without 20 µm Cd for 6 h and then were lysed with RIPA buffer at 4 °C. Next, the cell lysates were centrifuged to obtain the supernatants for further analysis. The MDA content, the activities of SOD and CAT, and the GSH/GSSG ratio were determined using the corresponding assay kits (all from Nanjing Jiancheng Bioengineering Institute, Nanjing, China) according to the instructions provided by the manufacturer.

*ROS Determination*: Cells grown in 96‐well plates were exposed to various doses of Cd for 6 h. DCF‐DA (Sigma, St. Louis, MO,USA) probe (10 × 10^−9^ m) was then added to the cells and incubated for an additional half hour. Finally, the generation of intracellular ROS was assessed by fluorescence analysis with an emission wavelength of 525 nm and an excitation wavelength of 488 nm on a microplate reader (Thermo Scientific).

*Statistical Analysis*: All data were analyzed by the Student's *t*‐test or one‐way analysis of variance test. Each experiment was performed at least three times, and the results were expressed as the mean +/− standard deviation (SD). *p*‐value less than 0.05 (*p < 0.05) or 0.001 (^#^ *p* < 0.001) indicated a statistically significant difference.

## Conflict of Interest

The authors declare no conflict of interest.
